# Function of the Golgi-located phosphate transporter PHT4;6 is critical for senescence-associated processes in Arabidopsis

**DOI:** 10.1093/jxb/erw249

**Published:** 2016-06-20

**Authors:** Sebastian Hassler, Benjamin Jung, Lilia Lemke, Ondřej Novák, Miroslav Strnad, Enrico Martinoia, H. Ekkehard Neuhaus

**Affiliations:** ^1^Plant Physiology, University of Kaiserslautern, Erwin-Schrödinger-Str., D-67653 Kaiserslautern, Germany; ^2^Laboratory of Growth Regulators, Centre of the Region Haná for Biotechnological and Agricultural Research, Palacký University & Institute of Experimental Botany ASCR, Šlechtitelů 11, CZ-78371 Olomouc, Czech Republic; ^3^Plant Biology, University of Zürich, Zürich, Switzerland

**Keywords:** Ammonium, cytokinin, Golgi, phosphate, salicylic acid, senescence, sugars.

## Abstract

The phosphate transporter PHT4;6 locates to the *trans*-Golgi compartment, and its impaired activity causes altered intracellular phosphate compartmentation, leading to low cytosolic Pi levels, a blockage of Golgi-related processes such as protein glycosylation and hemicellulose biosynthesis, and a dwarf phenotype. However, it was unclear whether altered Pi homeostasis in *pht4;6* mutants causes further cellular problems, typically associated with limited phosphate availability. Here we report that *pht4;6* mutants exhibit a markedly increased disposition to induce dark-induced senescence. In control experiments, in which *pht4;6* mutants and wild-type plants developed similarly, we confirmed that accelerated dark-induced senescence in mutants is not a ‘pleiotropic’ process associated with the dwarf phenotype. In fact, accelerated dark-induced senescence in *pht4;6* mutants correlates strongly with increased levels of toxic NH_4_^+^ and higher sensitivity to ammonium, which probably contribute to the inability of *pht4;6* mutants to recover from dark treatment. Experiments with modified levels of either salicylic acid (SA) or *trans*-zeatin (*t*Z) demonstrate that altered concentrations of these compounds in *pht4;6* plants act as major cellular mediators for dark-induced senescence. This conclusion gained further support from the notion that the expression of the *pht4;6* gene is, in contrast to genes coding for major phosphate importers, substantially induced by *t*Z. Taken together, our findings point to a critical function of PHT4;6 to control cellular phosphate levels, in particular the cytosolic Pi availability, required to energize plant primary metabolism for proper plant development. Phosphate and its allocation mediated by PHT4;6 is critical to prevent onset of dark-induced senescence.

## Highlight

Plants with altered intracellular Pi compartmentation surprisingly show accelerated dark-induced senescence. We propose that low cytosolic phosphate concentrations are of critical importance for proper plant development.

## Introduction

Phosphate is a macronutrient present in all types of cells, and its omnipresence is due to its requirement for synthesis of, for example, nucleic acids, various types of nucleotides, a large number of phosphorylated primary and secondary intermediates, or structural lipids necessary for functional biomembranes. Moreover, phosphate is involved in energy transfer, cellular signal transduction processes, and reversible protein modification, which further highlights the critical importance of this compound (Poirier and Bucher, 2002; Vance *et al.*, 2003).

Plants take up so-called inorganic phosphate (Pi) as HPO_4_^2–^ from the soil. In most soils, phosphate is limiting for plant growth and, as a constituent of fertilizers, Pi supply positively affects plant properties such as yield, speed of development, or tolerance against various biotic and abiotic stress stimuli (Bucher, 2007). Because of the importance of phosphate for most cellular processes, plants are able to sense cellular phosphate levels (Ticconi *et al.*, 2009) and modify their genetic program according to its availability (Müller *et al.*, 2007; Nakamura *et al.*, 2009).

Root cells import Pi from the soil via plasma membrane-located PHT1-type carriers, catalyzing a proton-coupled symport (Shin *et al.*, 2004). Inside plant cells, the unbound phosphate mainly locates to the largest organelle, the vacuole (Rausch and Bucher, 2002), which serves as a temporary store for a large number of different nutrients and metabolites (Martinoia *et al.*, 2007, 2012).

Transport of Pi across other cellular membranes is mediated by different transport proteins. While PHT1-type proteins represent a group of phosphate carriers usually found in the plasma membrane, the so-called PHT2-type carriers locate to the plastid envelope and PHT3-type carriers locate to the inner mitochondrial membrane (Rausch and Bucher, 2002; Weber *et al.*, 2005). Very recently, the carrier OsSPX-MFS3 from rice as well as the VPT1 protein from Arabidopsis (which are structurally not related to the other PHT proteins) have been identified as vacuolar Pi transporters (Liu *et al.*, 2015; Wang *et al.*, 2015). While OsSPX-MFS3 mediates Pi efflux from the vacuole to the cytosol, VPT1 is involved in the transport of Pi into the vacuole. A further, classical PHT type of phosphate transporter is the *trans*-Golgi-located PHT4;6 protein (Guo *et al.*, 2008; Hassler *et al.*, 2012) working as a transporter releasing Pi from the Golgi apparatus (Cubero *et al.*, 2009). Within the Golgi compartment, several ATP- and nucleotide sugar-dependent reactions release Pi, and PHT4;6 is thus required to allow export of Pi from the organellar lumen into the cytosol.

We recently showed that PHT4;6 loss-of-function mutants exhibit a dwarf growth and that they are compromised in both protein glycosylation and cell wall hemicellulose synthesis (Hassler *et al.*, 2012), representing processes typically associated with intact Golgi functions. Interestingly, PHT4;6 loss-of-function mutants show in addition increased pathogen defense properties associated with constitutively elevated levels of salicylic acid (SA) (Hassler *et al.*, 2012). Although the total phosphate content in *pht4;6* plants is similar to levels found in the correspondingly grown wild type, the mutants exhibit clear molecular symptoms of cellular Pi deficiency (Hassler *et al.*, 2012). This observation is due to altered intracellular phosphate compartmentation, leading to increased vacuolar Pi levels as determined by NMR spectroscopy (Hassler *et al.*, 2012).

Since cellular Pi starvation induce *inter alia* senescence-associated genes (SAGs; Wu *et al.*, 2003), since altered SA levels affect cellular senescence programs (Rivas-San and Plasencia, 2011; Vogelmann *et al.*, 2012), and because cellular vesicle transport (representing a process in which the Golgi apparatus is primarily involved) is associated with senescence (Yamazaki *et al.*, 2008), we took advantage of the properties of PHT4;6 to learn whether and how a disturbed phosphate homeostasis leads to an altered senescence program in Arabidopsis.

To study this process, we compared dark-induced senescence of the wild type and of PHT4;6 loss-of-function mutants. Dark incubation allows synchronization of onset of senescence (van der Graaff *et al.*, 2006), which can otherwise not be triggered simultaneously in the wild type and mutants, which exhibit, like *pht4;6* plants, a substantial developmental difference when compared with wild-type plants (Hassler *et al.*, 2012).

It turned out that *pht4;6* plants already show molecular symptoms of senescence during the standard day/night cycle and that dark-induced senescence is strongly accelerated in mutants, when compared with the wild type. Obviously, the function of PHT4;6 is critical to suppress senescence of dark-treated Arabidopsis plants. Alterations of SA and cytokinin metabolism in *pht4;6* plants seem to be involved in the modified senescence program of these mutants.

## Materials and methods

### Plant material and growth conditions

For all studies, *Arabidopsis thaliana* ecotype Col-0 and transgenic *pht4;6* plants (SAIL 809_B01, obtained from the Nottingham Arabidopsis Stock Centre; NASC) were used. PHT4;6 loss-of-function plants have already been described at the genetic and molecular level (Hassler *et al.*, 2012). *pht4;6* mutants overexpressing the *nahG* gene were created by transformation of the pCIB200-NahG vector (Syngenta, Basel, Switzerland) using the floral-dip method (Clough and Bent, 1998). Experiments on soil were performed on standard fertilized soil (ED-73) in a growth chamber (Weis-Gallenkamp) under short-day (SD) conditions (10h light/14h dark regime) at 21 °C and 125 µmol quanta m^−2^ s^−1^. For phosphate addition experiments, soil was watered with the same volume of either 25mM KH_2_PO_4_ solution or water. For dark treatment, plants were transferred into a box in the middle of the light period and incubated for the indicated times. For senescence recovery, experiments plants were incubated for up to 7 d in the dark prior to back-transfer to the given day/night cycle conditions. Liquid culture experiments were performed in Erlenmeyer flasks using half-strength Murashige and Skoog (1/2 MS; Duchefa) medium containing 0.05% MES with or without 1% sucrose. Plants used for nitrogen experiments were cultivated on agar plates with 1/2 MS medium (–N) with addition of KNO_3_, NaNO_3_, NH_4_Cl, or NH_4_PO_4_ as the sole nitrogen source. Seeds used for sterile culture were surface sterilized in 5% sodium hypochloride and subsequently incubated for 2 d in the dark at 4 °C for stratification.

### Gene expression analyses

Determination of mRNA abundance via northern blot was carried out as described previously (Jung *et al.*, 2011). DNA amplification for labeling probes was performed with the following primers: *SAG12*, SAG12_nor_fwd and SAG12_nor_rev; *SAG13*, SAG13_nor_fwd and SAG13_nor_rev; *NAC029*, NAP_nor_fwd and NAP_nor_rev; *SGN1*, NYE1_nor_fwd and NYE1_nor_rev; *ATG7*, ATAGP7_nor_fwd and ATAGP7_nor_rev; *S3H*, S3H_nor_fwd and S3H_nor_rev; and *WRKY53*, WRKY53_nor_fwd and WRKY53_nor_rev (see Supplementary Table S1 at *JXB* online). The RNA loading control was performed by ethidium bromide staining.

### Quantification of cytokinins

Samples for cytokinin measurements were ground under liquid nitrogen with a mortar and pestle, freeze-dried, and ground again with a Retsch ball-mill (MM301, http://www.retsch.de). Cytokinin extraction, purification, and quantification were carried out as described by Svačinová *et al.* (2012).

### Chlorophyll, protein, and RNA quantification

Chlorophyll quantification was performed according to a modified method (Arnon *et al*., 1949). A 50mg aliquot of ground plant material was dissolved in 80% (v/v) ethanol, heated for 10min at 95 °C, and centrifuged for 5min at 17 000 *g*. The supernatant was transferred and the extraction was repeated with the remaining sediment. Both supernatants were pooled and extinction was measured at 652nm in a microplate reader (Tecan Infinite 200 PRO, www.tecan.com).

Soluble proteins were isolated from 50mg of ground plant material by addition of 500 µl of phosphate-buffered saline (1× PBS; 137mM NaCl, 2.7mM KCl, 10mM Na_2_HPO_4_, 2mM KH_2_PO_4_, pH 7.4) supplemented with 10 µl ml^–1^ protease inhibitor (Sigma, P9599) and 1mM phenylmethylsulfonyl fluoride (PMSF). After centrifugation (5000 *g*, 5min, 4 °C), the supernatant was used for determination of soluble protein (Bradford, 1976). For SDS–PAGE analysis, 15 µg of protein was transferred to each lane. RNA isolation was performed as described earlier (Jung *et al.*, 2011). Concentrations of RNA were determined with a spectrophotometer (NanoDrop-1000, http://www.nanodrop.com).

### Quantification of soluble sugars and ammonium

For isolation of sugars and cations, plant material was ground under liquid N_2_, and 1ml of water was added to 100mg, mixed thoroughly, and heated for 15min at 95 °C. After centrifugation (15min, 18 000 *g*), the supernatant was used for ion chromatography quantification. Sugar quantification was performed by ion chromatography on a Metrosep CARB1-150 column (Metrohm, http://www.metrohm.com) using an 871-IC compact device (Metrohm) followed by amperometric quantification. NaOH (0.1M) was used as the mobile phase with a flow rate of 1ml min^–1^. The pressure was 9MPa at a temperature of 27 °C. Quantification of cations was performed by using a 761-IC compact device (Metrohm) on a Metrosep Cation column (Metrohm), followed by conductometry at a flow rate of 1ml min^–1^ and pressure of 7.5MPa, using 0.1N HNO_3_ and 1.7mM dipicolinic acid as the mobile phase.

### Determination of maximum quantum yield

The method was as described by Xiong *et al.* (2005). Plants were grown vertically on solid 1/2 MS agar containing 1% sucrose for 14 d. For analysis, the first and second true leaves were detached and incubated on 3MM-Whatman-paper soaked with 3mM MES solution (pH 5.7) in Petri dishes. Leaves were incubated in darkness for up to 6 d and the maximum quantum yield was analyzed with a chlorophyll fluorometer (Imaging-PAM M-series, MINI-version, Walz, Würzburg, Germany; http://www.walz.com) using ImagingWin software.

### Accession numbers

PHT4;6 (At5g44370), ATG7 (At5g45900), NAC029 (At1g69490), SGN (At4g22920), SAG12 (At5g45890), SAG13 (At2g29350), S3H (At4g10500), and WRKY53 (At4g23810).

## Results

### *pht4;6* mutant plants display an accelerated senescence phenotype induced by darkness

**Fig. 1.**
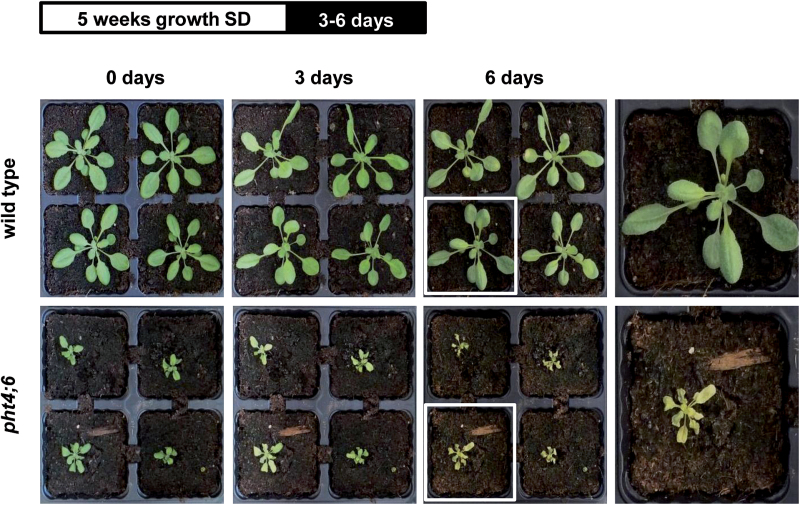
Dark-induced senescence in wild-type plants and *pht4;6* mutants. Plants were grown on soil for 5 weeks prior to incubation in darkness for the indicated time. Pictures were taken immediately after dark treatment. The top bar indicates a diagram of the growth conditions. Th right-hand panel shows a magnification of selected single plants after 6 d of darkness.

As stated above, *pht4;6* plants exhibit a number of altered cellular properties which might affect the plant senescence program. However, when grown on soil under standard SD conditions (10h light/14h dark), *pht4;6* mutant plants did not exhibit strong phenotypic symptoms of altered senescence when compared with wild-type plants (see Fig.1; left panel, 0 days). In contrast, incubation of both plant lines in the dark for 3 d resulted in a visible chlorosis in *pht4;6* mutants, while chlorosis could not be observed in wild-type plants (Fig. 1; 3 days). After 6 d of dark treatment, severe chlorosis in the mutant line was obvious and resulted in death of the plants. At this time point, the wild type showed only a few yellow spots, and most of the plants were still green and viable (Fig. 1; 6 days and right panel).

### Degradation of chlorophyll, proteins, and RNA is increased in the mutant line

**Fig. 2.**
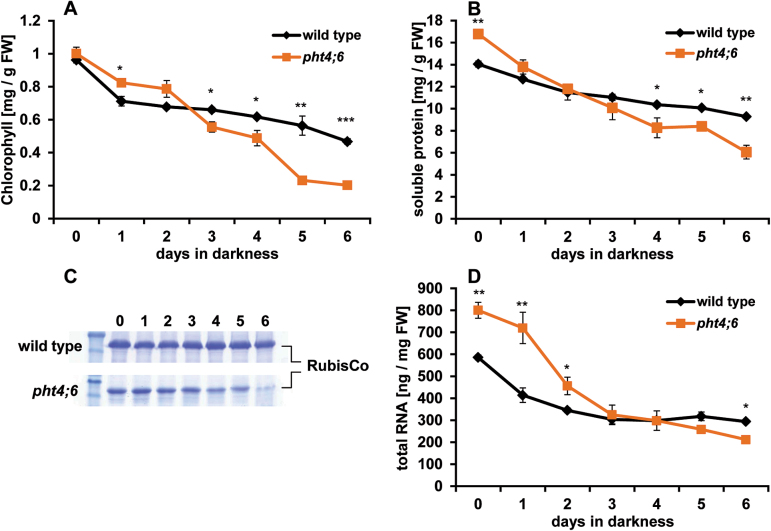
Dark-induced changes in chlorophyll, RNA, and protein levels. Wild-type plants and *pht4;6* mutants were grown for 5 weeks on soil followed by dark incubation for up to 6 d. (A) Chlorophyll content. (B) Content of soluble proteins. (C) SDS–PAGE visualizing degradation of RubisCO (LSU). Each slot was loaded with 15 µg of total soluble protein. (D) RNA levels. Error bars represent the SE of three biological replicates (*n*=3). Asterisks indicate statistically significant differences analyzed with Students *t*-test (*P≤0.05; **P≤0.01; ***P≤0.001).

The chlorophyll content of leaves can be used as an indicator to determine progression of plant senescence (Balazadeh *et al.*, 2008). To compare the course of dark-induced chlorophyll degradation in the wild type and *pht4;6* mutants, we grew corresponding plants for 5 weeks on soil under SD conditions, followed by incubation in the dark for up to 6 d (Fig. 2A). At each indicated time point, leaves from individual plants were harvested and chlorophyll contents were quantified.

At the beginning of the dark treatment, the chlorophyll contents in the wild type and *pht4;6* lines were nearly identical, 0.96±0.02mg g^–1^ FW and 1.00±0.04mg g^–1^ FW in the wild type and mutants, respectively (Fig. 2A). After 24h of dark treatment, a degradation of chlorophyll could be observed in both genotypes, and the wild type exhibited a slightly more reduced chlorophyll content (0.71±0.03mg g^–1^ FW) when compared with the mutant line (0.82±0.02mg g^–1^ FW, Fig. 2A). After 3 d in darkness, *pht4;6* plants showed a much stronger decrease of the chlorophyll content than the wild type, namely 0.56±0.03mg g^–1^ FW and 0.66±0.01mg g^–1^ FW, respectively. After 6 d in darkness, the chlorophyll content in *pht4;6* plants is only ~43% of the corresponding chlorophyll level observed in wild-type leaves (Fig. 2A).

During senescence, most of the leaf protein is mobilized by hydrolysis and as amino acids transported into developing sink tissues (Diaz *et al.*, 2008). Determination of soluble protein contents indicated that mutants contained at the beginning of the dark treatment ~20% more total protein (16.78±0.03 mg g^–1^ FW) than the corresponding wild type (14.04±0.28 mg g^–1^ FW; Fig. 2B). Induction of senescence by dark treatment resulted in a degradation of soluble proteins in both plant lines, but, as seen for chlorophyll (Fig. 2A), *pht4;6* plants also exhibited an accelerated decrease in the protein content when compared with the wild type (Fig. 2B). After 6 d of darkness, wild-type leaves still contained 9.28±0.06mg g^–1^ FW soluble protein, while mutants contained only 6.05±0.62mg g^–1^ FW. Visualization of the RubisCO content by SDS–PAGE in the wild type and mutants revealed that the latter genotype degraded this major leaf protein massively within 6 d of darkness, while the wild type is obviously able to keep the levels of this protein high (Fig. 2C).

Interestingly, *pht4;6* plants displayed ~37% higher total RNA contents at the beginning of the dark incubation, when compared with simultaneously grown wild-type plants (800.3±36.0ng g^–1^ FW and 585.7±12.0 nmg g^–1^ FW, respectively, Fig. 2D). After 3 d in the dark, RNA contents in both plants lines were decreased by 48% in wild-type plants and by 59% in mutant plants, respectively (Fig. 2D). During the next 3 d of darkness, total RNA in the wild type did not decrease further, while mutant plants degraded total RNA to 212ng g^–1^ FW, representing 72% of the corresponding wild-type level (Fig. 2D).

### *pht4;6* mutants show altered ammonium and sugar metabolism

For transport into sink tissues, most compounds present in plant leaves are mobilized during controlled senescence. The observation of a faster chlorophyll breakdown and of accelerated protein and RNA degradation in *pht4;6* plants (Fig. 2) led to the assumption that ammonium might accumulate in mutants when compared with the situation in wild-type plants.

**Fig. 3.**
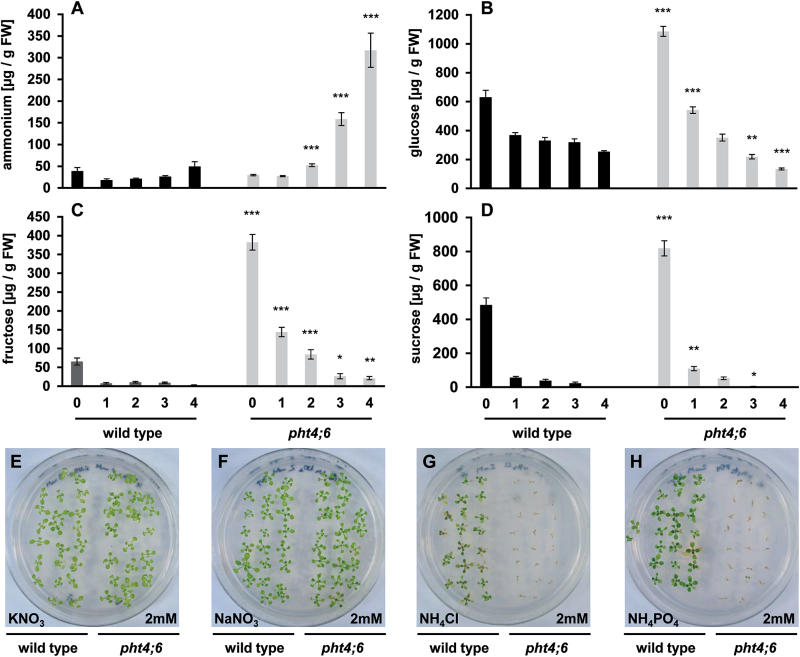
Quantification of ions and carbohydrates in dark-incubated plants and growth phenotype of plants cultivated on different nitrogen sources. (A–D) Wild-type plants and *pht4;6* mutants were grown for 5 weeks on soil followed by dark incubation for up to 4 d. (A) Ammonium contents. (B) Glucose levels. (C) Fructose levels. (D) Sucrose levels. (E–H) Plants were cultivated for 2 weeks on agar plates containing 1/2 MS medium (–N) with the indicated nitrogen sources. (E) KNO_3_ (2mM) as nitrogen source. (F) NaNO_3_ (2mM) as nitrogen source. (G) NH_4_Cl as nitrogen source. (H) NH_4_PO_4_ as nitrogen source. Error bars indicate the SE of eight individual replicates (*n*=8). Asterisks indicate statistically significant differences analyzed with Students *t*-test (*P≤*0.05; **P≤0.01; ***P≤0.001).

For corresponding analyses, we determined the ammonium levels in both plant lines under SD conditions and during dark-induced senescence for up to 4 d (Fig. 3A). During growth in the standard light/dark cycle, the plant lines did not differ statistically in their ammonium levels (the wild type contained 38.84±8.27 µg g^–1^ FW ammonium and *pht4;6* contained 29.63±1.50 µg g^–1^ FW ammonium, respectively; Fig. 3A). However, after only 2 d in the dark, the first significant differences in the ammonium levels between the two plant lines were obvious: *pht4;6* plants contained 52.33±3.13 µg g^–1^ FW ammonium while the wild type contained only 21.25±1.57 μg g^–1^ FW (Fig. 3A). This difference was further increased within the next 2 d of dark incubation and resulted in an accumulation of 317.16±39.5 µg g^–1^ FW ammonium in mutant plants and only 49.46±11 µg g^–1^ FW in the wild type.

Interestingly, under standard growth conditions, *pht4;6* plants contain increased levels of glucose, fructose, and sucrose when compared with the wild type (Fig. 3B–D). Both glucose and sucrose are found to be ~1.7-fold more abundant in *pht4;6* than in wild-type leaves, while fructose levels are ~6-fold increased in mutant leaves (Fig. 3B–D). During dark incubation, all three types of sugars declined rapidly in both genotypes (Fig. 3B–D), indicating a massive carbon consumption, presumably by oxidative phosphorylation to maintain metabolic activity. Due to the comparable high levels of glucose, fructose, and sucrose in *pht4;6* mutants, the relative loss of sugars within the first 4 d in this plant line is substantially higher than in the corresponding wild type (Fig. 3B–D).

Obviously, *pht4;6* plants accumulate relatively large amounts of toxic NH_4_^+^ after onset of senescence (Fig. 3A). To analyze whether these mutants show general difficulties in withstanding NH_4_^+^, we grew wild-type and *pht4;6* plants in the presence of either nitrate or ammonium. In the presence of nitrate as the sole nitrogen source, both wild-type and *pht4;6* plants exhibited the same growth efficiency (Fig. 3E, F). In contrast, in the presence of ammonium (given either as the chloride or the phosphate salt) *pht4;6* mutants were unable to develop, while wild-type plants developed similarly to the development in the presence of nitrate as the sole nitrogen source (Fig. 3G, H).

### *pht4;6* mutants are unable to recover after dark incubation and show specific changes in ammonium and sugar metabolism

**Fig. 4.**
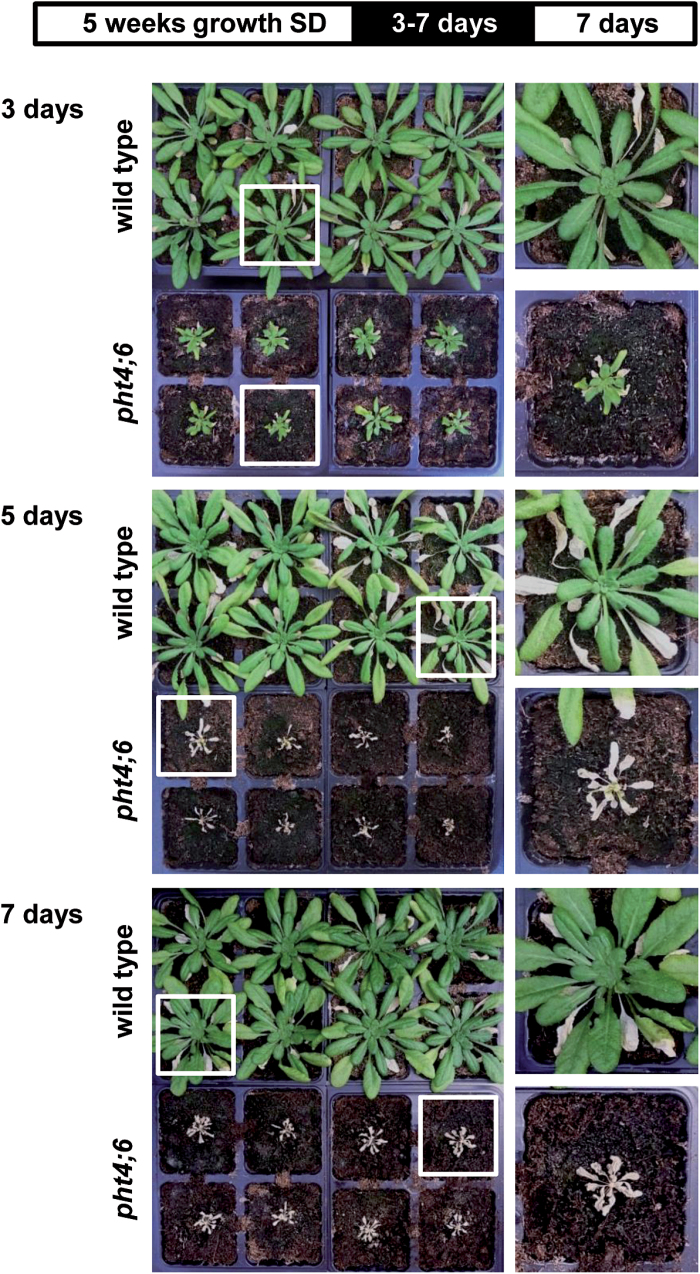
Recovery of wild-type plants and *pht4;6* mutants after dark incubation. Plants were grown on soil for 5 weeks and were then incubated in darkness for the indicated time. After either 3, 5, or 7 d of dark incubation, recovery in the light was allowed for 7 d. The top bar indicates a diagram of the growth conditions. Insets show magnifications of selected single plants.

Since *pht4;6* mutants exhibit accelerated induction of dark-induced senescence (Figs 1, 2), we were interested to discover whether this mutant shows decreased ability to restore the dark-induced senescence program. For this analysis, we incubated both plant lines for 3–7 d in darkness and transferred them, subsequent to this senescence trigger phase, back into the light (Fig. 4).

Wild-type plants which have been incubated for either 3, 5, or 7 d in darkness recovered (within 7 d in SD conditions) completely and showed only a few chlorotic and dead leaves (Fig. 4). This result is in marked contrast to the performance of *pht4;6* plants, since the latter exhibit no recovery after >3 d of permanent darkness (Fig. 4). Already after 5 d of darkness, all *pht4;6* leaves appeared chlorophyll free and completely dried out (Fig. 4).

**Fig. 5.**
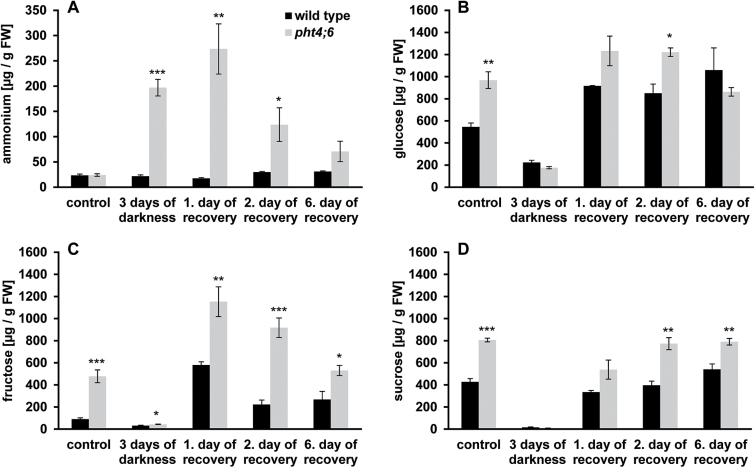
Determination of metabolites during the recovery of wild-type plants and *pht4;6* mutants. Plants were grown on soil for 5 weeks and were then incubated in darkness for 3 d. Subsequently, recovery in the light was allowed for up to 6 d. Metabolites were analyzed before and during the recovery process. (A) Determination of ammonium. (B) Determination of glucose. (C) Determination of fructose. (D) Determination of sucrose. All plants were harvested in the middle of the light period. Error bars represent the SE of four biological replicates (*n*=4). Asterisks indicate statistically significant differences analyzed with Students *t*-test (*P≤*0.05; **P≤0.01; ***P≤0.001).

During the recovery process, we analyzed the corresponding leaf ammonium and sugar contents. Under standard growth conditions, both plant lines contained similar levels of ammonium, namely ~24 µg g^–1^ FW (Fig. 5A). However, in contrast to the wild type, *pht4;6* mutants contain significantly increased ammonium levels during both dark induction (Fig. 3A) and subsequent re-illumination (Fig. 5A). After 1 d of re-illumination, *pht4;6* plants contained ~15-fold higher ammonium levels than the corresponding wild type, namely 273.6 µg g^–1^ FW and 17.6 µg g^–1^ FW ammonium, respectively (Fig. 5A). Similarly to ammonium, *pht4;6* plants accumulate higher levels of sugars (glucose, fructose, and sucrose) during onset of re-illumination (Fig. 5B–D; see also Fig. 4B–D). These differences are most pronounced after 1 d of re-illumination, when *pht4;6* plants contained 1.3-fold increased glucose, 2-fold increased fructose, and 1.6-fold increased sucrose when compared with wild-type levels (Fig. 5B-D).

### Altered salicylic acid and *trans*-zeatin metabolism in *pht4;6* plants is involved in accelerated senescence

**Fig. 6.**
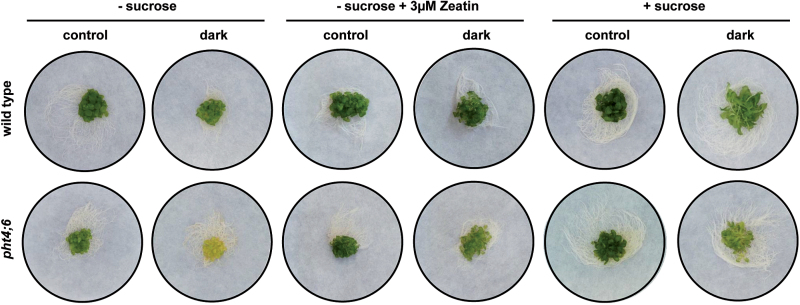
Dark-induced senescence in wild-type plants and *pht4;6* mutants grown in liquid culture. Twenty seedlings of each line were grown for 2 weeks in 1/2 MS supplemented with 1% sucrose under short-day conditions. Subsequent to this, the growth medium was changed to 1/2 MS without sucrose, 1/2 MS without sucrose+3 µM *trans*-zeatin, or 1/2 MS with 1% sucrose. Plants were incubated in the dark for one additional week. Control lines were not incubated in the dark. Experiments were repeated three times with similar results.

**Fig. 7.**
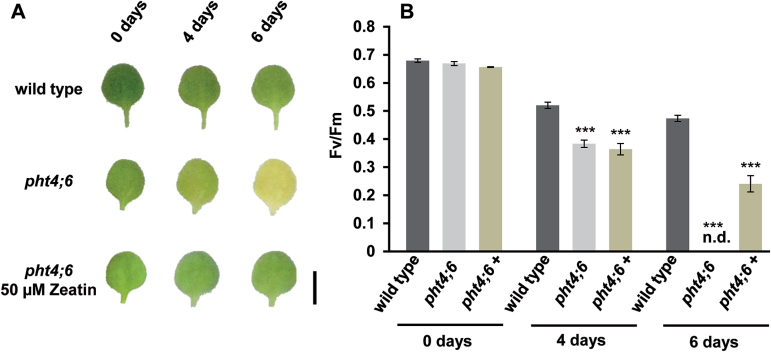
Effect of dark incubation (± *trans*-zeatin) on photosynthetic parameters. Wild-type and *pht4;6* seedlings were grown under short-day conditions on vertical agar plates (1/2 MS+1% sucrose) for 2 weeks. The first and second true leaves were detached and incubated in the dark on 3MM-Whatman-paper soaked with 3mM MES solution (pH 5.7, ± *trans*-zeatin) for the indicated time. (A) Phenotypic appearance of wild-type and *pht4;6* leaves during dark incubation. Scale bar=0.5cm. (B) Maximum quantum yield of dark-incubated leaves. Error bars represent the SE of eight leaves (*n*=8). n.d. (not detectable), no chlorophyll fluorescence was detectable. Experiments were repeated twice with similar results. Asterisks indicate statistically significant differences analyzed with Students *t*-test (***P≤0.001).

We have already shown that *pht4;6* mutants exhibit a dwarf phenotype with reduced shoot biomass (Hassler *et al.*, 2012). To reveal whether the accelerated senescence in this mutant is due to the small size of the plants, we conducted further growth experiments under either liquid culture conditions or on agar plates. These two growth conditions prevent the appearance of a dwarf phenotype at least in the early developmental phase (Figs 6, 7).

However, even under conditions of liquid culture, *pht4;6* plants showed accelerated dark-induced senescence after 3 d in darkness (Fig. 6). Remarkably, the additional presence of *trans*-zeatin (*t*Z) prevented senescence symptoms in *pht4;6* mutants, and also the application of sucrose strongly suppressed senescence in these mutants (Fig. 6).

When grown on agar plates, the average sizes of wild-type and mutant leaves are very similar (Fig. 7A). However, dark induction again provoked a rapid senescence of *pht4;6* plants (Fig. 7A), while wild-type plants stayed green (Fig. 7A). Thus, even under conditions in which *pht4;6* plants do not phenotypically differ from the wild type, an accelerated senescence is obvious in mutants. Again, application of *tZ* prevents onset of senescence in *pht4;6* plants (Fig. 7A). This zeatin-caused absence of senescence in mutants is fully confirmed by corresponding measurements of photosynthesis. At 6 d post-onset of dark incubation, the presence of zeatin still allowed photosynthetic activity of *pht4;6* mutants, as revealed by an *F*_v_/*F*_m_ value of ~0.27 (Fig. 7B), while in the absence of *tZ* no fluorescence was measurable, indicating that the photosynthetic machinery is destroyed (Fig. 7B).

We have already reported that *pht4;6* plants exhibit substantially increased levels of SA. Some bacteria contain an SA-specific hydroxylase encoded by the gene *NahG*. The NahG protein thus degrades SA and has frequently been used to decrease endogenous SA levels in plants (Friedrich *et al.*, 1995). We stably introduced the corresponding gene under control of the *Cauliflower mosaic virus* 35S promoter into the *pht4;6* mutant to check for the effect of SA (Supplementary Fig. S1).

**Fig. 8.**
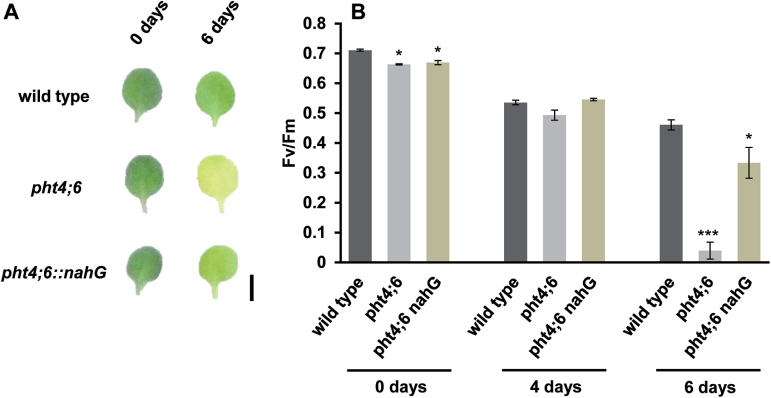
Effect of dark incubation on photosynthetic parameters. Wild-type, *pht4;6*, and *pht4;6::nahg* seedlings were grown under short-day conditions on vertical agar plates (1/2 MS+1% sucrose) for 2 weeks. The first and second true leaves were detached and incubated in the dark on 3MM-Whatman-paper soaked with 3mM MES solution for the indicated time. (A) Phenotypic appearance of wild-type and *pht4;6* leaves during dark incubation. Scale bar=0.5cm. (B) Maximum quantum yield of dark-incubated leaves. Error bars represent the SE of 16 leaves (*n*=16). Experiments were repeated three times with similar results. Asterisks indicate statistically significant differences analyzed with Students *t*-test (*P≤*0.05; ***P≤0.001).

*pht4;6::nahg* mutants exhibited fewer symptoms of dark-induced senescence when compared with *pht4;6* plants (Fig. 8A). Even after 6 d in darkness, the photosynthetic efficiency, which is completely abolished in *pht4;6* mutants (see Fig. 7B), in *pht4;6::nahg* plants is similar to ratios observed in the wild type (Fig. 8B; Supplementary Fig. S2).

### Senescence-associated genes as well as autophagy- and SA-responsive genes display altered expression in the *pht4;6* mutant

The expression of a large number of genes has to be altered to induce senescence in plants. These SAGs encode, for example, proteases, transcription factors, proteins involved in mobilization of nitrogen and carbon, or transport proteins (Buchanan-Wollaston *et al.*, 2003; Guo *et al.*, 2004). Prominent genes used for detection of aging in leaves are, for example, the late senescence gene *SAG12*, encoding a cysteine protease, or *SAG13*, coding for an oxidoreductase expressed in the early phase of senescence.

**Fig. 9.**
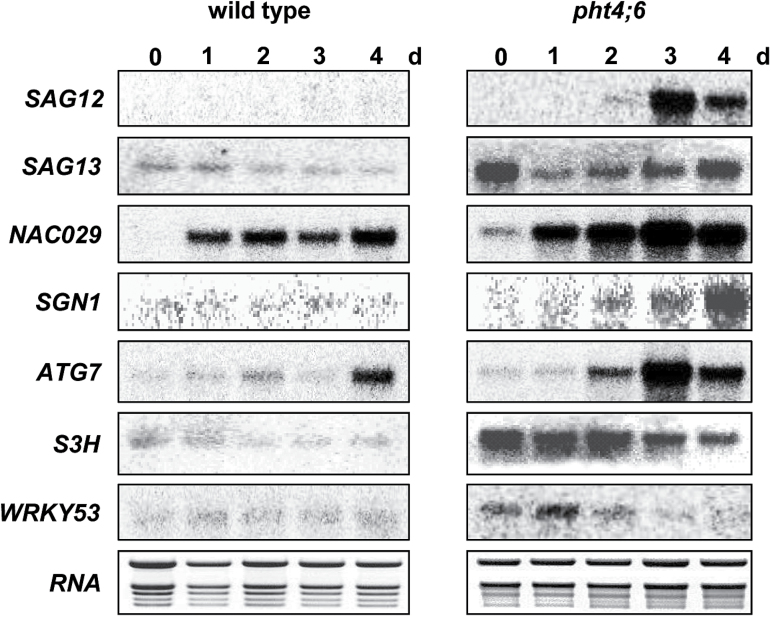
Gene expression analysis of wild-type plants and *pht4;6* mutants during dark incubation. Tissue of individual plants was collected either in the middle of the light period (0 d) or at the same time (14:00h) before and during dark treatment for up to 4 d and analyzed with ^32^P-labeled probes for *SAG12*, *SAG13*, *NAC029*, *SGN1*, *ATG7*, *S3H*, and *WRKY53*. A total of 5mg of RNA was used in each lane. Ethidium bromide staining of RNA was used as a loading control.

During dark treatment, RNA radiolabeling (northern blot analysis) revealed that the *SAG12* gene is not induced in the wild type, while high expression could be detected in *pht4;6* mutants after just 3 d, followed by a decrease of transcript at day four (Fig. 9). Surprisingly, the expression of *SAG13* was already high under standard growth conditions in the mutants (Fig. 9) and decreased markedly after 1 d of dark treatment. Subsequent to this, the *SAG13* transcript increased slightly again (Fig. 9). *SAG13* mRNA was also slightly detectable in wild-type plants but, similar to the situation in *pht4;6* plants, the expression during dark incubation decreased from day to day (Fig. 9). *NAC029*, coding for a senescence-induced transcription factor, already showed a stronger expression in *pht4;6* plants before dark treatment when compared with the wild type (Fig. 9). The transcript level of *SGN1*, coding for a protein involved in chlorophyll degradation (Park *et al.*, 2007), accumulated in mutants after the fourth day of darkness, while no *SGN1* mRNA was detectable in the wild-type plants (Fig. 9).

Autophagy is a process that is induced to extend survival under unfavorable conditions, such as nutrient starvation or prolonged darkness (Doelling *et al.*, 2002; Yoshimoto *et al.*, 2004). For transcriptional analysis of autophagy, we analyzed *ATG7* mRNA. A weak *ATG7* expression was detectable in the wild type until day three of dark treatment, and at day four mRNA accumulated strongly in these plants (Fig. 9). In *pht4;6* plants, *ATG7* mRNA is extremely abundant at day three of dark treatment.

S3H, a salicylic acid 3-hydroxylase, is responsible for the *in planta* conversion of SA to 2,3- or 2,5-dihydroxybenzoic acid. Interestingly, *S3H* transcripts were constitutively increased in *pht4;6* plants, when compared with wild-type plants, and decreased during dark treatment (Fig. 9). The low constitutive *S3H* mRNA level in the wild type further decreased after onset of dark-induced senescence (Fig. 9). The transcription factor *WRKY53* contributes to the regulation of the endogenous SA concentration and plays an important role in activation of senescence (Hinderhofer and Zentgraf, 2001). In *pht4;6* plants, *WRKY53* mRNA is constitutively higher than in the wild type and declines after onset of senescence (Fig. 9). In wild-type plants, the low level of *WRKY53* mRNA under standard growth conditions is only slightly increased by dark treatment (Fig. 9).

### Zeatin contents are altered in the *pht4;6* line

Cytokinins represent hormones with a superior impact on aging, since high cytokinin levels prevent onset of senescence (Gan and Amasino, 1995). Given that *pht4;6* plants show accelerated dark-induced senescence, it was thus of importance to check for alterations in cytokinin metabolism in this mutant plant.

**Fig. 10.**
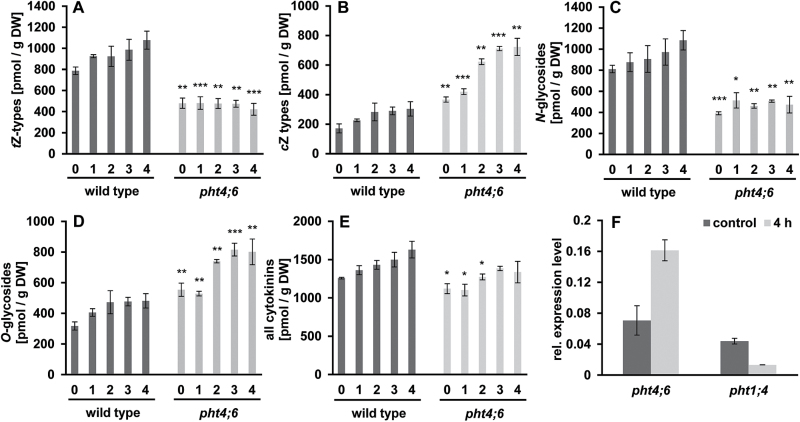
Quantification of cytokinins (and their derivatives) in wild-type plants and *pht4;6* mutants, and quantitative analysis of *PHT* gene expression in response to *trans*-zeatin. (A) Determination of total *trans*-zeatin types before and during dark incubation. (B) Determination of total *cis*-zeatin types before and during dark incubation. (C) Determination of the *N*-glycoside content before and during dark incubation. (D) Determination of the *O*-glycoside content before and during dark incubation. (E) Quantification of all cytokinins in wild-type and *pht4;6* plant tissue before and during dark incubation. (F) Relative expression of the *PHT4;6* and *PHT1;4* genes in wild-type plants after 4h *trans*-zeatin feeding (1mM); *UBQ10* was used as the reference gene. Error bars represent the SE of three plants (*n*=3). Asterisks indicate statistically significant differences analyzed with Students *t*-test (*P≤*0.05; **P≤0.01; ***P≤0.001).

For this, we quantified levels of *t*Z and *cis*-zeatin (*c*Z), as well as dihydrozeatin (DHZ), *N* ^6^-(Δ^2^-isopentenyl)adenine (iP), and their glycosylated derivatives (Fig. 10). When grown under SD conditions, wild-type plants contained significantly higher *t*Z levels (787.42±35.88 pmol g^–1^ DW) when compared with *pht4;6* plants (479.64±48.45 pmol g^–1^ DW). After onset of dark-induced senescence, *t*Z in wild-type plants further increased, reaching 1077.36±85.83 pmol g^–1^ DW, while *t*Z contents in *pht4;6* plants remained low (421.85±56.17 pmol g^–1^ DW; Fig. 10). For the less active cytokinin type *c*Z, the levels were the opposite. The wild type accumulated 171.35±30.17 pmol g^–1^ DW *c*Z when grown under a standard day/night cycle, while *c*Z in *pht4;6* plants was >2-fold higher, namely 366.73±17.35 pmol g^–1^ DW (Fig. 10). Wild-type plants accumulated only slightly more *c*Z until the fourth day of dark treatment (303.5±48.55 pmol g^–1^ DW), but in *pht4;6* plants *c*Z concentrations increased further, reaching 722.71±57.99 pmol g^–1^ DW (Fig. 10). We also quantified the levels of conjugated zeatins (*N*- and *O*-glycosides; Fig 10C, D) and observed a significantly reduced content of *N*-glycosides in *pht4;6* plants before (390.78±14.47 pmol g^–1^ DW) and after the fourth day of dark treatment (473.37±79.02 pmol g^–1^ DW) compared with the wild type (811.25±34.96 pmol g^–1^ DW and 1083.96±92.03 pmol g^–1^ DW). In contrast, the concentrations of the *O*-glycosides were significantly increased in mutant plants (Fig. 10D). This could be observed before and during dark incubation. Overall, the total cytokinin content in *pht4;6* plants compared with the wild type was reduced (Fig. 10E).

In addition, we have analyzed the transcriptional response of the *PHT1;4* and *PHT4;6* gene after zeatin feeding in wild-type plants (Fig. 10). Interestingly, while the expression of *PHT1;4* is decreased after zeatin feeding (Sakakibara *et al.*, 2006), the expression pattern of the *PHT4;6* gene behaves in an opposite way and increases.

### The addition of phosphate results in a delayed dark-induced senescence in *pht4;6* plants

**Fig. 11.**
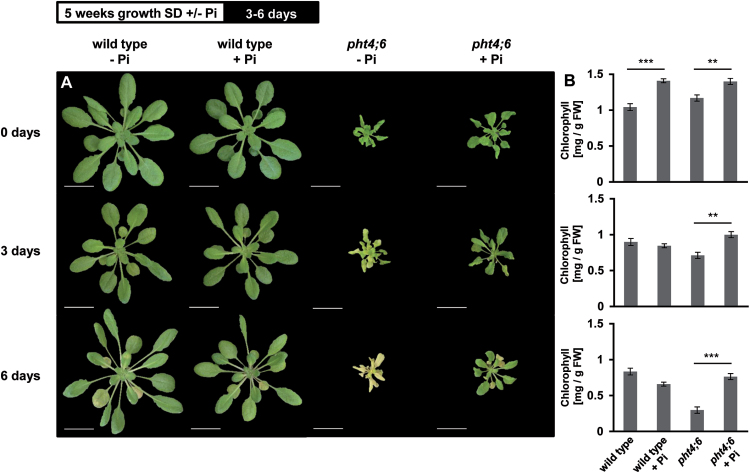
Dark-induced senescence of wild-type plants and *pht4;6* mutants with and without supplement of 25mM phosphate to the soil. (A) Plants were grown on soil for 5 weeks prior to incubation in darkness for the indicated time. Pictures were taken immediately after dark treatment. Scale bar=2cm. (B) Chlorophyll content of dark-treated plants. Error bars represent the SE of four plants (*n*=4). Experiments were repeated twice with similar results. The top bar indicates a diagram of the growth conditions. Asterisks indicate statistically significant differences analyzed with Students *t*-test (**P≤0.01; ***P≤0.001).

Previously we showed that the addition of 25mM phosphate to the soil stimulates growth of the *pht4;6* mutants (Hassler *et al.*, 2012). To analyze if an improved phosphate availability can also prevent the onset of senescence in *pht4;6* plants, we added 25mM phosphate to the water supplied daily for 5 weeks. Subsequent to this, we incubated both genotypes in darkness for up to 6 d (Fig. 11A). Phosphate-limiting conditions (–Pi) resulted in a reduced growth of *pht4;6* plants when compared with growth in the presence of additional Pi (Fig. 11A). In contrast, growth efficiency of wild-type plants is not affected by additional Pi administration (Fig. 11A, upper panel). Quantification of chlorophyll revealed that improved phosphate availability results in an increased content of this pigment in both wild-type and *pht4;6* plants (Fig. 11B). Three days after onset of darkness, the first chlorosis could be detected in *pht4;6* plants grown without additional Pi, while the presence of additional Pi prevented chlorosis (+/– Pi; Fig. 11A). After 6 d of dark incubation, *pht4;6* mutants displayed severe chlorosis and showed pale, yellow leaves while wild-type (–Pi), and phosphate-supplemented wild-type and *pht4;6* plants showed only a few yellow leaves (Fig. 11A). This effect is also obvious when quantifying the chlorophyll content of the different plant lines (Fig. 11B). After 6 d in darkness, *pht4;6* plants showed a decrease of Chl to <25% when compared with the initial content (Fig. 11B). In contrast, the additional presence of phosphate during growth resulted in a dark-induced decrease of chlorophyll to only 55%, which is comparable with similar effects in wild-type plants (+Pi values).

## Discussion

It has been shown on cell suspension cultures as well as on intact plants that nutrient starvation is a trigger of stress and often results in senescence (Auberts *et al.*, 1994; Crafts-Brandner *et al.*, 1998). Accordingly, Pi deficiency can induce a molecular senescence response (Wu *et al.*, 2003). Similarly to published results, *pht4;6* plants also display a substantial transcriptional increase of genes typically related to Pi starvation (Lei *et al.*, 2011; Hassler *et al.*, 2012). These results indicate that senescence is related not only to phosphate starvation, but also to a disturbed phosphate homeostasis, since the mutant and wild-type plants contain similar total intracellular levels of Pi.

PHT4;6 deletion mutants exhibit an impaired phosphate homeostasis due to impaired Pi release from the Golgi compartment. We were therefore interested in analyzing whether impaired Pi release from the Golgi compartment might lead to accelerated senescence in this mutant line. To this end, we decided not to induce senescence-related processes by aging because wild-type and *pht4;6* mutants differ substantially in their phenotypic appearance (Hassler *et al.*, 2012; Fig. 1), and several reports show that mutants exhibiting an impaired development show altered senescence when compared with wild-type plants (Dai *et al.*, 1999; Baena González *et al.*, 2007; Yoshimoto *et al.*, 2009). Instead we decided to induce senescence by transferring wild-type and mutant plants into continuous darkness. This approach has been shown to be a standardized procedure leading to highly reproducible results (Kunz *et al.*, 2009; Jung *et al.*, 2011). Nevertheless, a distinction has to be made between starvation-, developmental-, and dark-induced senescence. All three forms of senescence exhibit different gene expression patterns as well as specific metabolic changes (Buchanan-Wollaston *et al.*, 2005). Since we do not observe an earlier occurring developmental senescence in the *pht4;6* mutant, we focused on dark-induced senescence.

### Accelerated senescence of *pht4;6* mutants is caused by cytosolic Pi starvation and not by impaired plant development

When compared with wild-type plants, *pht4;6* mutants indeed exhibit a substantially accelerated senescence when transferred into continuous darkness (Fig. 1). This conclusion is based on the observations that upon transfer into darkness, *pht4;6* plants exhibit a severe yellowing of leaves and a rapid decrease of chlorophyll, total protein, total RNA, and sugar contents (Figs 1, 2). This loss of critical cellular components is typically associated with cellular reprogramming during senescence (Buchanan-Wollaston *et al.*, 2003), representing conditions in which transfer of nutrients such as nitrogen (stored in proteins or chlorophyll), phosphate (stored in nucleic acids), or carbon (stored in sugars) from leaves to seeds or storage organs (e.g. roots) takes place (Himelblau and Amasino, 2001; Hörtensteiner and Feller 2002; Robinson *et al.*, 2012).

In a previous study, we demonstrated, that *pht4;6* plants show molecular symptoms of Pi deficiency, although their total intracellular levels of inorganic phosphate are high (Hassler *et al.*, 2012). Since apparently the low cytosolic Pi leads to accelerated senescence, we fed the mutant and wild-type plants with an excess of Pi (25mM) with the goal of increasing the cytosolic Pi concentration. This treatment abolished the dark-induced accelerated senescence in *pht4;6* plants and led to nearly identical levels of chlorophyll in wild-type and mutants leaves after prolonged darkness (Fig. 11). These observations suggest that lack of cytosolic Pi in *pht4;6* plants is responsible for fast induction of the senescence program and that Pi accumulated within the Golgi cannot be recycled rapidly through the secretory pathway.

The observation that *pht4;6* plants supplemented with Pi senesce similarly to the wild type (Fig. 11) while the dwarf phenotype is not rescued indicates that the faster senescence in the mutant plants is not a ‘simple’ pleiotropic reaction associated with impaired plant development and that the dwarf phenotype is more likely to be due to a general disturbance of the Pi homeostasis and not only due to a too low cytosolic Pi concentration. While in the PHT4;1 loss-of-function mutant (Karlsson *et al.*, 2015) defects and retarded plant growth were caused by the limited availability of Pi for stromal ATP synthesis which could also be recovered by additional phosphate supply (Karlsson *et al.*, 2015), impaired PHT4;6 function apparently leads to a more complex defect in Pi homeostasis.

The assumption that the dwarf phenotype and the accelerated senescence in *pht4;6* mutants are processes that are not directly related gains further support by the analysis of mutants grown under either liquid culture conditions or on agar plates. Under these cultivation conditions, the supplemented phosphate concentration seems to be sufficient to delay the early appearance of a *pht4;6*-related dwarf phenotype but could not prevent it completely. Nevertheless, also under these conditions, an accelerated dark-induced senescence could be observed (Figs 6, 7).

### *pht4;6* mutants exhibit a primed cellular senescence program which is repressed by photosynthesis

Comparison of SAG expression in wild-type and *pht4;6* plants (Fig. 9) clearly showed that a large number of SAGs are more highly expressed in mutants than in the corresponding wild-type plants. Remarkably, not only are the mRNAs deriving from late senescence-associated genes (e.g. *SAG12*, *NAC029*, or *ATG7*; Doelling *et al.*, 2002; Guo and Gan, 2006) more abundant in mutants after 1–4 d in darkness, but also that of the early senescence-associated gene *SAG13* (Lohman *et al.*, 1994; Fig. 9). We propose that the high mRNA level of this early SAG may indicate that in mutant plants senescence occurs very rapidly and is already initiated during the short dark period during the night but that this short period does not lead to onset of the complete cellular senescence program under the periodic day/night conditions. A prolonged exposure to darknes would then really also initiate visible and physiological processes leading to rapid senescence that cannot be reversed, as observed in Fig. 4.

We attribute this significantly increased sensitivity of mutant plants to darkness to at least two factors. First, *pht4;6* plants, in contrast to wild-type plants, accumulate much higher levels of NH_4_^+^ (Fig. 3A). However, NH_4_^+^, as a product of protein and chlorophyll degradation (Peeters and van Laere, 1992; Chen *et al.*, 1997; Masclaux *et al.* 2000), acts generally as an agent uncoupling electrochemical gradients across biomembranes, leading to substantial toxicity. Secondly, *pht4;6* plants mobilize their high endogenous sugar levels during the entire dark phase much more efficiently than wild-type plants, leading to virtually no free sucrose after just 3 d of darkness (Fig. 3D). That this lack of sucrose in dark-treated *pht4;6* plants supports senescence symptoms in *pht4;6* plants is substantiated by data from plants after feeding of sucrose, since additional sucrose feeding substantially delays loss of chlorophyll (Fig. 6).

Ammonia fixation during amino acid synthesis takes places in chloroplasts via glutamine synthase coupled to the enzyme GOGAT (Masclaux-Daubresse *et al.*, 2010). We demonstrated that photosynthesis in particular is negatively affected in dark-treated *pht4;6* plants (Fig. 7A, B) and it thus seems likely that NH_4_^+^ re-assimilation during onset of light after a period of dark is impaired in mutants when compared with wild-type plants. This assumption gained experimental support from the quantification of both NH_4_^+^ and sugars in wild-type and mutant plants after 3 d of dark treatment. While wild-type plants keep the level of toxic NH_4_^+^ low during both darkness and subsequent re-illumination, NH_4_^+^ in *pht4;6* leaves even increased after the first photoperiod (Fig. 5). The latter observation is indicative of a blockage in chloroplast-associated ammonia fixation. In fact, the nearly complete inability of *pht4;6* mutants to develop in the presence of NH_4_^+^ (Fig. 3G, H) supports our view that the markedly high ammonium levels in mutants after dark incubation contributes to their overall increased sensitivity to dark-induced senescence. This high level of ammonia even seems to prevent induction of the essential metabolic recovery, because newly synthesized sugars, which obviously have to derive from residual photosynthesis, cannot become as efficiently drained into anabolic reactions in *pht4;6* plants, when compared with the situation in the wild type (Fig. 5B–D).

### Accelerated senescence in *pht4;6* plants is caused by altered Pi homeostasis but signaled via salicylic acid and zeatin

Some other Arabidopsis loss-of-function plants, such as mutants lacking the autophagy-related gene *ATG5* (Yoshimoto *et al.*, 2009) or the *MILDEW RESISTANCE LOCUS O2* (*MLO2*; Consonni *et al.*, 2006) display similarities to *pht4;6* in respect to (i) altered SA contents; (ii) expression of pathogen-related genes; and (iii) accumulation of H_2_O_2_ (Hassler *et al.*, 2012). Interestingly, these two mutants also exhibit an early leaf senescence phenotype (Consonni *et al.*, 2006; Yoshimoto *et al.*, 2009).

It is well known that mutants with altered cell wall composition show a modified pathogen resistance (for a review, see Miedes *et al.*, 2014). In *pht4;6* plants, the hemicellulose content is affected; furthermore, they exhibit an enhanced resistance against *Pseudomonas syringae* and display an increased level of SA (Hassler *et al.*, 2012). Whether this SA accumulation is caused by an altered cell wall composition is not yet known. However, for *Medicago sativa* mutants with a reduced lignin content, it was hypothesized that SA is a central component in response to secondary cell wall integrity, and the SA accumulation observed might be a result of activation of endogenous defense responses by elicitor-active polysaccharides (Gallego-Giraldo *et al.*, 2011). Nevertheless, a constitutively activated pathogen defense may cause accumulation of SA, which is, in addition to its role in plant–pathogen interaction, a well-known inducer of senescence (Morris *et al.*, 2000). Under normal day/night growth, *pht4;6* plants presumably compensate this effect by both conjugation of SA to sugars and the conversion of SA to dihydroxybenzoic acids, mediated by the enzyme S3H (Zhang *et al.*, 2013). The latter assumption receives support from the observation that the *S3H* mRNA levels are extremely high in *pht4;6* mutants (Fig. 9), which might represent an endogenous reaction of the mutant to constitutively enhanced SA levels (Hassler *et al.*, 2012). In any case, since we showed that a degradation of SA in the *pht4;6* mutant—by the introduction of the bacterial salicylate hydroxylase gene (*nahG*; Friedrich *et al.*, 1995; Supplementary Fig. S1)—delays onset of dark-induced senescence (Fig. 8), we conclude that SA is one inducer of senescence in *pht4;6* plants.

Besides SA, other cellular compounds have additional impact on onset of the plant senescence program (Schippers *et al.*, 2007; Jibran *et al.*, 2013). In this context, we mention in particular *tZ*, which belongs to the diverse group of natural cytokinins (for a detailed review, see Kieber and Schaller 2014). Early studies already provided evidence that plant senescence can be delayed either by addition of cytokinins (Noodén *et al.*, 1979) or by increased endogenous *tZ* synthesis (Gan and Amasino, 1995). Surprisingly, even before onset of darkness, we detected an ~40% decreased content of *tZ* derivatives in *pht4;6* plants when compared with wild-type plants. These compounds, in contrast to *cZ* derivatives (which highly accumulate in mutant plants; Fig. 10), represent cytokinins with high biological activity (for details, see Sakakibara, 2006).

To explain the low levels of *tZ* in *pht4;6* mutants (Fig. 10A), we have to mention that cytokinins interact with phosphate signaling in Arabidopsis, leading to a low cytokinin concentration under conditions of Pi starvation (Franco-Zorrilla *et al.*, 2005). So it appears likely that the impaired cellular Pi homeostasis leading to starvation syndromes in *pht4;6* mutants (Hassler *et al.*, 2012) is a driving signal causing low *tZ* contents (Fig. 10). The observation that exogenous application of *tZ* results in a repression of dark-induced senescence (Figs. 6, 7) supports our conclusion that low *tZ* levels and onset of senescence is not a pleiotropic reaction in *pht4;6* plants.

Zeatin addition experiments have shown that cytokinins repress many genes induced by phosphate starvation, including the phosphate transporter genes *PHT1;2* and *PHT1;4* (Martín *et al.*, 2000; Sakakibara *et al.*, 2006; Fig. 10). In marked contrast, the *PHT4;6* gene was up-regulated after zeatin feeding, indicating that zeatin acts as an upstream regulator of *PHT4;6* expression involved in regulation of intracellular Pi homeostasis.

To understand this specific effect, we have to consider that *tZ*, in general, stimulates plant development and growth. Thus, an adaptation of intracellular Pi availability seems to be a prerequisite to complete zeatin-induced processes. The cause is likely to be that high metabolic activity is linked to a high need for Pi, for example for continuing ATP regeneration, synthesis of phosphorylated intermediates and lipids, as well as for RNA and DNA synthesis. In fact, the observation that *pht4;6* plants accumulate large amounts of neutral sugars and have a low capacity for NH_4_^+^ re-fixation, when compared with the wild type (Fig. 5A–D), indicates cellular difficulties in this mutant to energize primary metabolism. Taken together, our data on the interaction of altered intracellular Pi homeostasis and *tZ* signaling are fully in line with previous observations on zeatin signaling mutants. As described by Franco-Zorilla *et al.* (2005), a close crosstalk between these metabolites exists since impaired *tZ* sensing in *ahk4* (*cre1, wol1*) and *ahk2/ahk3* mutants also results in enhanced sugar sensitivity and an impact on the Pi starvation response. In other words, we here provide the first evidence that intracellular Pi homeostasis, rather than the total cellular Pi level, governs zeatin-specific processes in Arabidopsis.

## Supplementary data

Supplementary data are available at JXB online

Figure S1. Analysis of *pht4;6::nahG* overexpression plants.

Figure S2. Chlorophyll fluorescence image of the analyzed plants.

Table S1. List of primers used in this work.

Supplementary Data

## Abbreviations:

cZ: *cis*-zeatin
DHZ: dihydrozeatin
iP: *N* ^6^-(Δ^2^-isopentenyl)adenine
SA: salicylic acid
SAG: senescence-associated gene
SD: short day
tZ: *trans*-zeatin.
