# Supplementary material for: Function of the Golgi-located phosphate transporter PHT4;6 is critical for senescence-associated processes in Arabidopsis
Source: J Exp Bot. 2016 Jun 20;67(15):4671–84. doi: 10.1093/jxb/erw249 (PMC4973741; doi:10.1093/jxb/erw249)
Supplement: Supplementary Data [file supp_67_15_4671__index.html]

Function of the Golgi-located phosphate transporter PHT4;6 is critical for senescence-associated processes in Arabidopsis — Function of the Golgi-located phosphate transporter PHT4;6 is critical for senescence-associated processes in Arabidopsis — Supplementary Data 

# Function of the Golgi-located phosphate transporter PHT4;6 is critical for senescence-associated processes in Arabidopsis

## Supplementary Data

Data files

- supplementary\_figures\_S1\_S2\_table\_S1.pdf - Supplementary Data
