# Supplementary material for: Function of the Golgi-located phosphate transporter PHT4;6 is critical for senescence-associated processes in Arabidopsis
Source: J Exp Bot. 2016 Jun 20;67(15):4671–84. doi: 10.1093/jxb/erw249 (PMC4973741; doi:10.1093/jxb/erw249)
Supplement: Supplementary Data [file supp_erw249_supplementary_figures_S1_S2_table_S1.pdf]

Function of the Golgi-located phosphate transporter PHT4;6 is critical for senescence associated processes in Arabidopsis

Sebastian Hassler, Benjamin Jung, Lilia Lemke, Ondrej Novak, Miroslav Strnad, Enrico Martinoia, and Ekkehard H. Neuhaus

Supplemental File

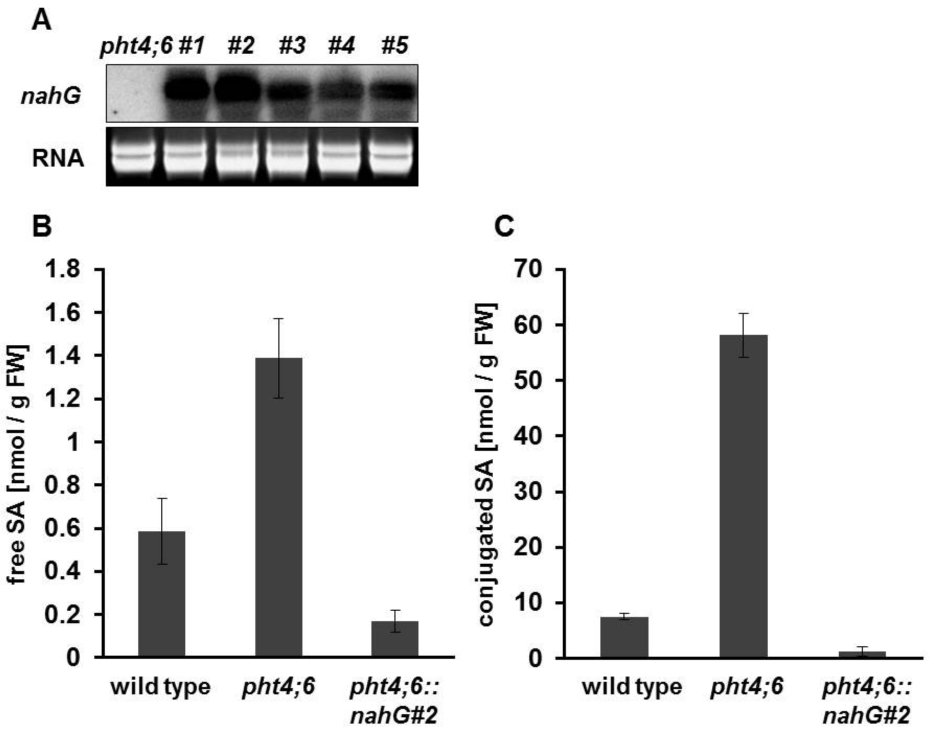

Fig. S1. Analysis of *pht4;6::nahG* overexpression plants. (A) Transcript analysis of *pht4;6* mutants, overexpressing the *NahG* gene. (B) Quantification of free salicylic acid in five week old wild-type, *pht4;6* and *pht4;6::nahG* plants. (C) Quantification of conjugated salicylic acid in five week old wild-type, *pht4;6* and *pht4;6::nahG* plants. Error bars represent standard error of three plants (n=3).

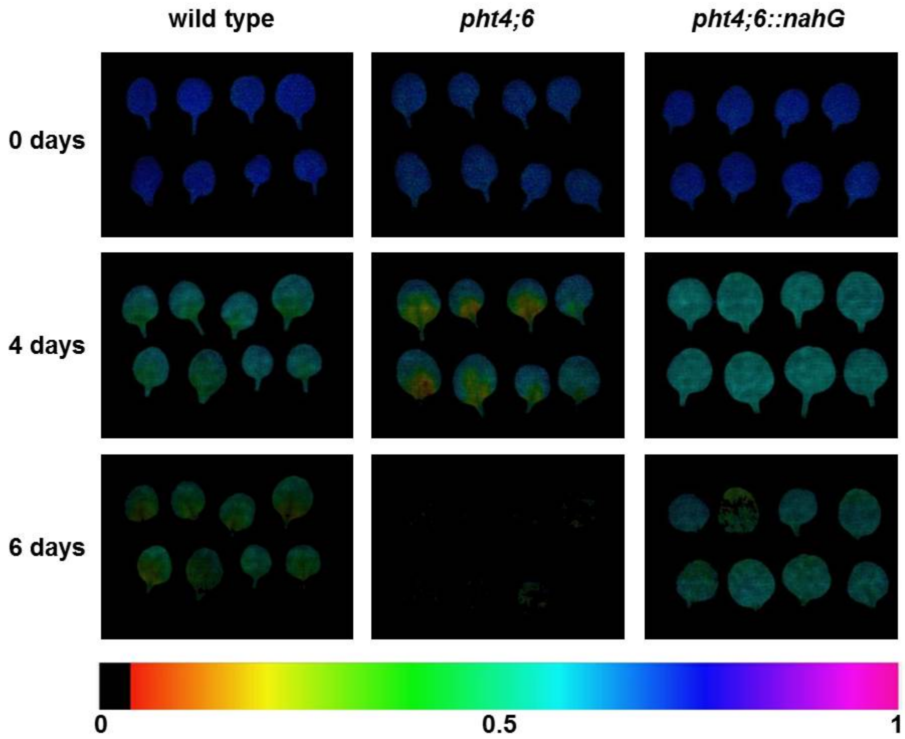

Fig. S2. Chlorophyll fluorescence image of wild-type, *pht4;6* and *pht4;6::nahG* plants. Leaves of plants were detached after 2 week growth on vertical ½MS agar plates and incubated in the dark for the indicated time. The analyses of Fv/Fm ratio was performed with dark adapted plants. Images are normalized to the false colour bar below.

Table S1: Primers used for amplification of DNA and for quantitative analyses by RT-PCR.

Primer	Sequence (5' - 3')
SAG12_nor_fwd	CTCGTCCACTCGACAATGAAC
SAG12_nor_rev	GGGTAAGAAGCTTTCATGGC
SAG13_nor_fwd	GCAACCAAAGGAGCCATG
SAG13_nor_rev	GTTTGGCCAACTAGTCTGC
NAP_nor_fwd	GGAAGTAACTTCCCAATCTACC
NAP_nor_rev	AACATCGCTTGACGATGATGG
NYE1_nor_fwd	GAGATGTGTAGTTTGTCGGCG
NYE1_nor_rev	CTAGAGTTTCTCCGGATTTGGAG
ATAGP7_nor_fwd	GGGATGGGAGCTTAACAAAG
ATAGP7_nor_rev	CATCAGTATCATCGTCTTCCC
S3H_nor_fwd	GATTTCGCCTCCTCCGTTC
S3H_nor_rev	CTCAACAAACGGGTAGGTTC
WRKY53_nor_fwd	GGAAGAGATATGTTAAGTTGGG
WRKY53_nor_rev	GGGAAAGTTGTGTCAATCTCG
nahG-sense	GCCTTAGCACTGGAACTCTG
nahG-antisense	TCGGTGAACAGCCACTTGCAC
PHT1;4_RT_fwd	TCAATGGCGTTGCCTTCTGT
PHT1;4_RT_rev	ATCACCAAGCCACCCGAAA
PHT4;6_RT_fwd	TTCCTCAGTGGCTCTTGGG
PHT4;6_RT_rev	AGATACAGAGCAAACCCGGG
UBQ10_RT_fwd	GGCCTTGTATAATCCCTGATGAATAAG
UBQ10_RT_rev	AAAGAGATAACAGGAACGGGAAACATAGT
